# Knowledge, Attitude, and Practice of the Medical Students of the University of Medical Sciences and Technology Towards Hepatitis B Virus Infection

**DOI:** 10.7759/cureus.88402

**Published:** 2025-07-21

**Authors:** Layla M Babiker, Ahmed I Idriss, Elmustafa I Idriss, Fatima Elzahra Kheirelsid

**Affiliations:** 1 Internal Medicine, Africa International University (AIU), Cairo, EGY; 2 Internal Medicine, Sudan International University, Khartoum, SDN; 3 Internal Medicine, Faculty of Medicine, University of Khartoum, Khartoum, SDN; 4 Internal Medicine, Faculty of Medicine, University of Medical Sciences and Technology, Khartoum, SDN; 5 Internal Medicine, University of Khartoum, Khartoum, SDN

**Keywords:** attitude, hepatitis b, knowledge, practices, university of medical sciences and technology

## Abstract

Background

Hepatitis B virus (HBV) infection is a serious global health concern, particularly for healthcare workers and medical students who are at elevated occupational risk due to their clinical exposure. Despite the availability of effective vaccines, knowledge gaps and suboptimal prevention practices persist.

Objective

To assess the knowledge, attitudes, and practices (KAP) of medical students at the University of Medical Sciences and Technology in Sudan regarding HBV infection.

Methods

A descriptive, cross-sectional study was conducted among 235 medical students in their third to fifth years. Data were collected using a validated, self-administered questionnaire. Statistical analysis was performed using Statistical Product and Service Solutions (SPSS, version 26; IBM SPSS Statistics for Windows, Armonk, NY). Descriptive statistics were calculated, and associations were tested using chi-square, Kruskal-Wallis, and Spearman’s rank correlation. A p-value of <0.05 was considered statistically significant.

Results

Of the 235 participants, 54.9% demonstrated good knowledge of HBV, 68.1% had a fair attitude, while 56.2% showed poor preventive practices. Significant associations were found between knowledge and age (p=0.007), occupation (p<0.001), and source of information (p=0.014). Gender significantly influenced practice levels (p=0.033). Positive correlations were observed between knowledge and attitude (p<0.001), knowledge and practice (p=0.001), and attitude and practice (p<0.001).

Conclusion

Although most students were knowledgeable and held fair attitudes toward HBV, a large proportion failed to adopt appropriate preventive practices. Mandatory vaccination, clinical training reinforcement, and awareness campaigns are recommended to improve student preparedness and reduce occupational HBV risks.

## Introduction

Hepatitis B virus (HBV) infection remains a significant global public health concern [1]. Approximately 10% of infected individuals develop chronic hepatitis, and 15%-25% progress to cirrhosis or hepatocellular carcinoma [2,3]. HBV is recognized as a major infectious occupational hazard for healthcare workers, including medical students, due to their repeated exposure to blood and body fluids during clinical training [1,4]. The risk is particularly high among trainees because of their limited experience, insufficient training, and lack of awareness regarding preventive measures [5].

Importantly, HBV infection is one of the most preventable causes of chronic liver disease [4]. The hepatitis B vaccine is the most effective and safe strategy for prevention [5]. Enhancing public understanding of the signs, symptoms, and transmission routes of HBV is critical for limiting disease spread. Consequently, numerous studies have examined the knowledge, attitude, and practice (KAP) of healthcare students regarding HBV infection [6-8].

The World Health Organization (WHO) recommends HBV vaccination for individuals at occupational risk, including healthcare professionals and students. However, despite the high exposure risk, global HBV vaccination coverage among healthcare workers remains suboptimal [7]. In Sudan, where HBV prevalence is moderate to high, there is a scarcity of data evaluating the awareness and practices of medical students toward HBV prevention [3,8]. This study aims to assess the KAP of medical students at the University of Medical Sciences and Technology (UMST) regarding HBV and highlight the importance of vaccination in this high-risk group.

Problem statement

HBV infection can lead to chronic liver disease, cirrhosis, or hepatocellular carcinoma, posing a severe threat to global health. Healthcare professionals and medical students are particularly vulnerable due to their occupational exposure. Despite this elevated risk, WHO reports indicate low HBV vaccination coverage among healthcare workers worldwide, including students [7]. Previous studies conducted in various countries have reported inadequate knowledge, attitudes, and practices (KAP) regarding HBV among medical students, especially concerning transmission routes and prevention measures.

Justification

This study contributes to the national effort to reduce HBV-related morbidity in Sudan by evaluating medical students' awareness and behavior toward the disease. While several international studies have explored KAP regarding HBV among healthcare students, limited data are available from Sudan. By assessing the KAP of UMST medical students, this study seeks to fill a crucial gap in the literature and inform future public health interventions targeted at healthcare trainees in the country.

## Materials and methods

Study design

An institutional-based cross-sectional study was conducted at the UMST using the data collected from a questionnaire distributed to, and filled by, the third- to fifth-year medical students.

Study area

The study was done at the UMST. The university is located in Al Riyadh, Khartoum State, in Sudan.

Study population

The study population consisted of third- to fifth-year medical students enrolled at the UMST during the study period.

Inclusion criteria

Medical students in the third, fourth, or fifth academic year who were enrolled at the university and selected as part of the random sample.

Sample size

The sample size was estimated through the equation n=N/1+Nd2, where n is the estimated sample size, N is the total number of questionnaires filled by the medical students, and d is the degree of accuracy set at 0.05. The samples were collected during the period from March 2024 to June 2024. Using the above equation, the sample size was found to be 235.

Sampling technique

The randomized sampling technique was used to get a sample of the questionnaires to be filled out by the third- to fifth-year medical students of the UMST.

Data collection technique

Data were collected through a data collection sheet filled by the researcher from questionnaires distributed to and filled by the third- to fifth-year medical students of the UMST from the period between the 1st of March and 31st of June 2024. The datasheet was validated by a senior professional specialized in research methodology.

Data analysis and management

The collected data, which were retrieved from the collection sheet, were exported into Microsoft Excel and then imported into the Statistical Product and Service Solutions (SPSS, version 26; IBM SPSS Statistics for Windows, Armonk, NY). Descriptive percentage was calculated by (Frequency * 100 / Sample size) and was presented in tables and figures. Chi-square test, Kruskal-Wallis H test, and Spearman's rank correlation were used to assess the significant association. P value is significant when it is < 0.05.

Interpretation

The categorization of KAP levels was based on participants’ scores. For knowledge, scores ranging from 0 to 5 were classified as poor, 6 to 10 as fair, and 11 to 15 as good. Attitude levels were categorized as poor for scores between 0 and 8, fair for scores from 9 to 16, and good for scores from 17 to 24. Similarly, practice scores from 0 to 1 indicated poor practice, 2 to 3 indicated fair practice, and scores between 4 and 5 were considered good practice.

## Results

Sociodemographic data

Out of 235 students in this study (Table 1), females were the most represented (51.5%, n=121), and more than half of them were between 21 and 23 years old (54.9%, n=129). Additionally, most participants were fifth-year medical students (33.6%, n=79).

**Table 1:** Sociodemographic data of the participants in the study (n=235) Descriptive statistics are presented as N and percentage (%). No inferential test applied.

Sociodemographic Data		Count	Valid N %
Gender	Female	120	51.5%
	Male	115	48.9%
Age	17-20	92	39.1%
	21-23	129	54.9%
	24-26	14	6.0%
Occupation	3^rd^ year	78	33.2%
	4^th^ year	78	33.2%
	5^th^ year	79	33.6%

Source of information about hepatitis

**Figure 1:**
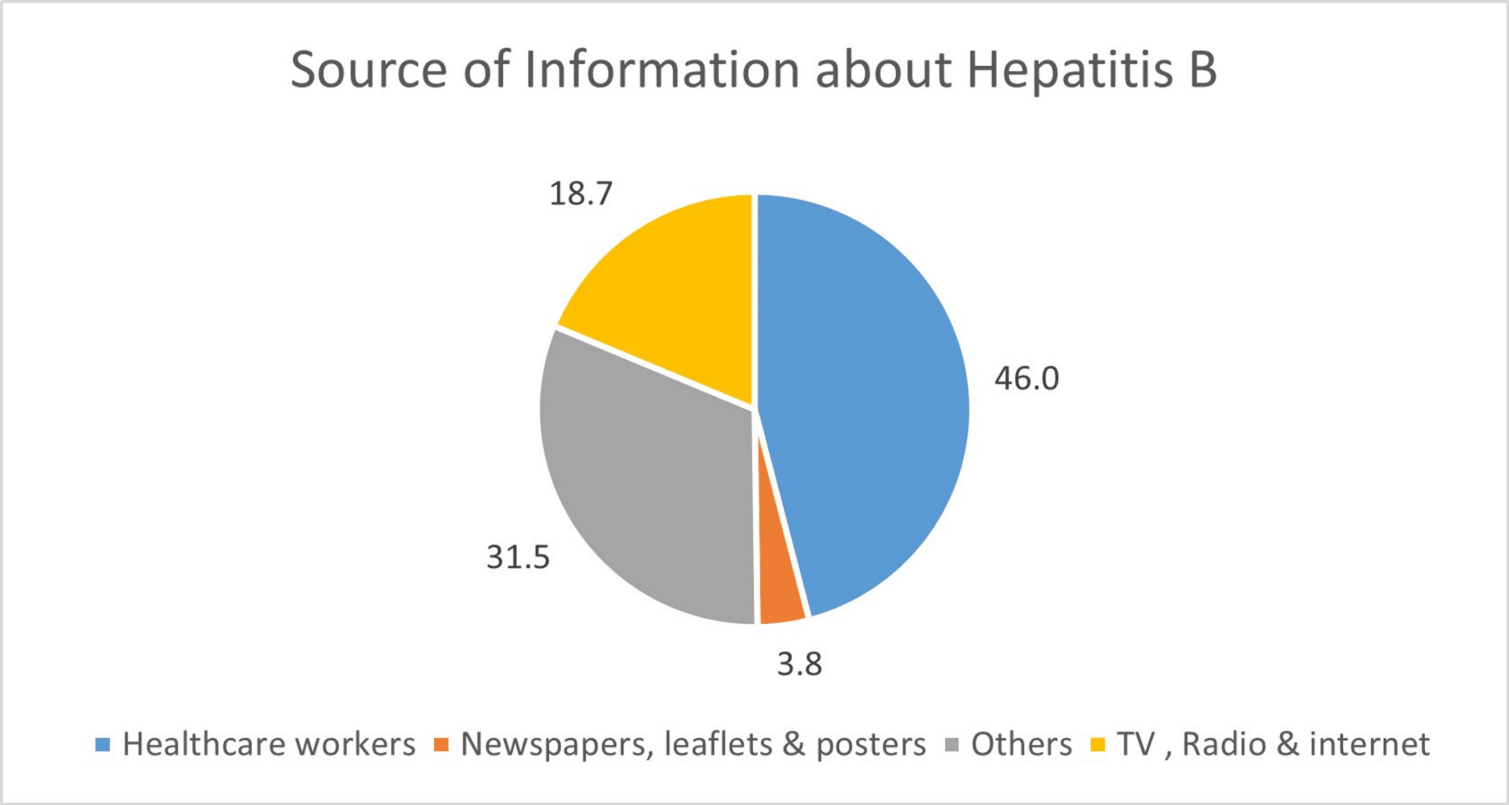
Source of information about hepatitis B among participants (n=235) Data are presented as percentages (%) of participants (n=235).

The majority of participants (46.0%, n = 108) reported obtaining information from healthcare workers (Figure 1). Other sources included newspapers, leaflets, and posters (3.8%, n=9), various unspecified sources categorized as "Others" (31.5%, n=74), and TV, radio, and the internet (18.7%, n=44).

Assessment of knowledge of hepatitis B

The majority correctly identified that hepatitis B is transmitted through sharing needles and syringes (90.6%, n=213) and contaminated blood and blood products (85.5%, n=201) (Table 2). A significant portion also recognized that it can be transmitted by sharing used blades in barbershops or ear/nose piercing tools (63.8%, n=150).

**Table 2:** Knowledge about transmission methods of hepatitis B among participants (n=235) Data are presented as frequencies (N) and percentages (%).

Transmission Method	Frequency	Percent
Contaminated blood and blood products	201	85.5%
Sharing needles and syringes	213	90.6%
Shaking hands	6	2.6%
Sharing used blades of barbers or ear and nose piercing tools	150	63.8%
Coughing, sneezing	6	2.6%
Sharing food with an infected person	15	6.4%
Mother to baby transmission	130	55.3%
Unsafe sex	179	76.2%
Drinking water	3	1.3%
I don’t know	2	0.9%

The most commonly identified symptoms included jaundice (87.7%, n=206), abdominal pain (78.7%, n=185), vomiting (65.5%, n=154), and nausea (46.8%, n=110) (Table 3).

**Table 3:** Knowledge about the common symptoms of hepatitis B among participants (n=235) Data are presented as frequencies (N) and percentages (%).

Symptom	Frequency	Percent
Excessive hunger	6	2.6%
Sweating	122	51.9%
Vomiting	154	65.5%
Abdominal pain	185	78.7%
Excessive talking	3	1.3%
Nausea	110	46.8%
Jaundice	206	87.7%
Loss of appetite	134	57.0%
I don't know	13	5.5%

Most participants (82.6%, n=194) correctly identified that hepatitis B can cause liver cancer (Table 4). Only 45.5% (n=107) believed the disease is curable. Regarding vaccination, a substantial majority (93.6%, n=220) recognized that a vaccine is available. Most participants (66.0%, n=155) knew that an asymptomatic HBV-positive individual can transmit the virus. Finally, 66.8% (n=157) correctly understood that sharing eating or drinking utensils does not transmit hepatitis B.

**Table 4:** Knowledge about various aspects of hepatitis B among participants (n=235) Data are presented as frequencies (N) and percentages (%).

Question	Yes	No	I Don’t Know
Does hepatitis B cause liver cancer?	194 (82.6%)	12 (5.1%)	29 (12.3%)
Is hepatitis B a curable disease?	107 (45.5%)	53 (22.6%)	75 (31.9%)
Is there any vaccine for hepatitis B?	220 (93.6%)	4 (1.7%)	11 (4.7%)
If a person confirmed as hepatitis B virus positive (+ ve), but doesn’t present any signs and symptoms, do you think that person can transmit hepatitis B virus?	155 (66.0%)	43 (18.3%)	37 (15.7%)
Does sharing, eating, and drinking utensils transmit hepatitis B?	20 (8.5%)	157 (66.8%)	58 (24.7%)

Assessment of attitude towards hepatitis B

**Figure 2:**
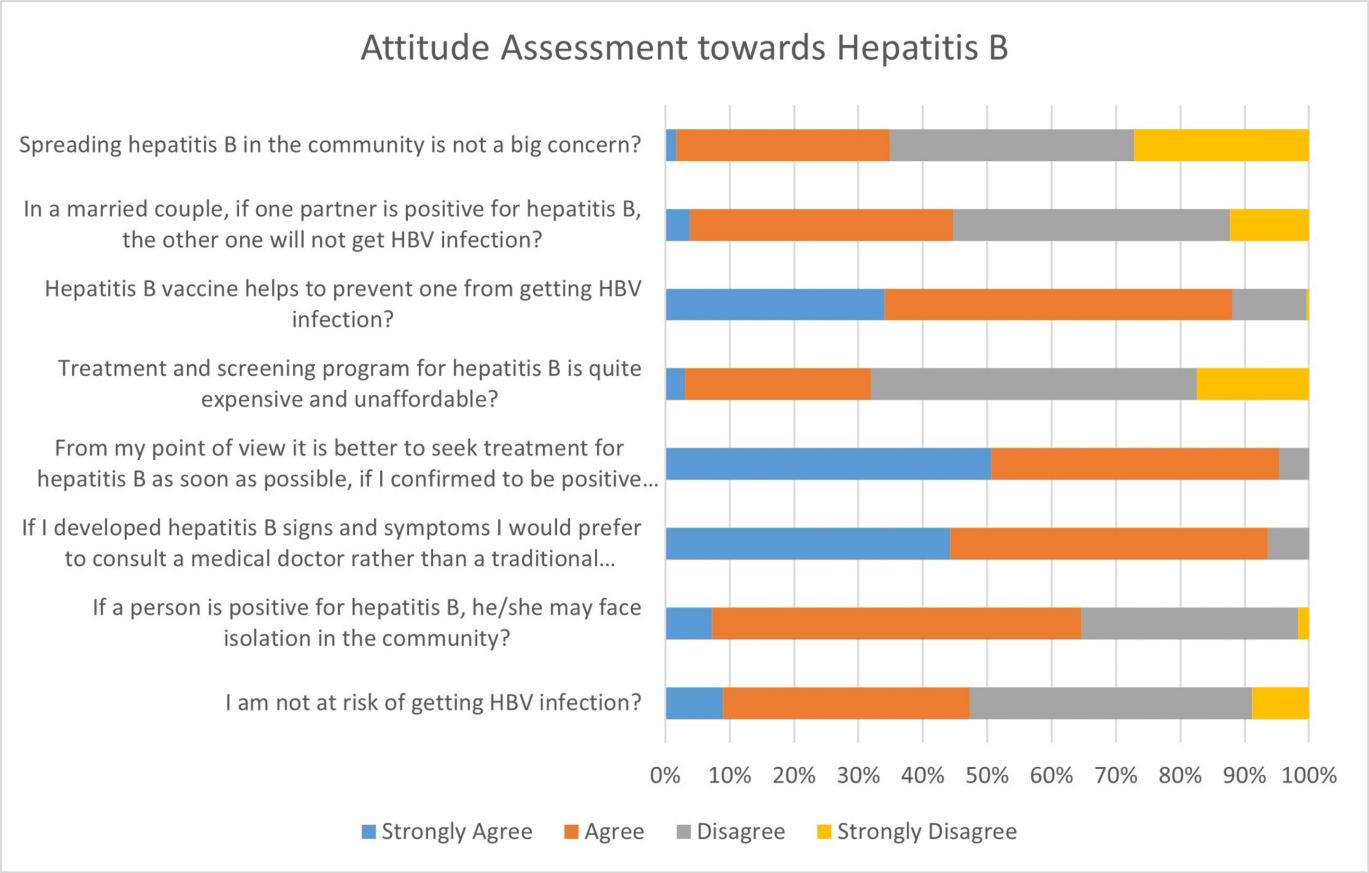
Attitude assessment towards hepatitis B among participants (n=235) Data are presented as percentages (%) of responses to attitude statements (n=235).

Most participants (47.2%, n=111) believed that they are at risk of HBV infection, and 64.6% (n=152) noted that HBV-positive individuals may face social isolation (Figure 2). A preference for consulting medical doctors was expressed by 93.7% (n=220), and 95.0% (n=223) favored early treatment upon diagnosis. Concerns about cost were shared by 31.9% (n=75), and 88.0% (n=207) acknowledged the HBV vaccine’s effectiveness. Regarding HBV transmission between spouses, 44.7% (n=105) believed that the uninfected partner would not contract it. Meanwhile, 34.9% (n=82) believed that HBV spread is not a major community concern.

Assessment of the practice of hepatitis B

**Figure 3:**
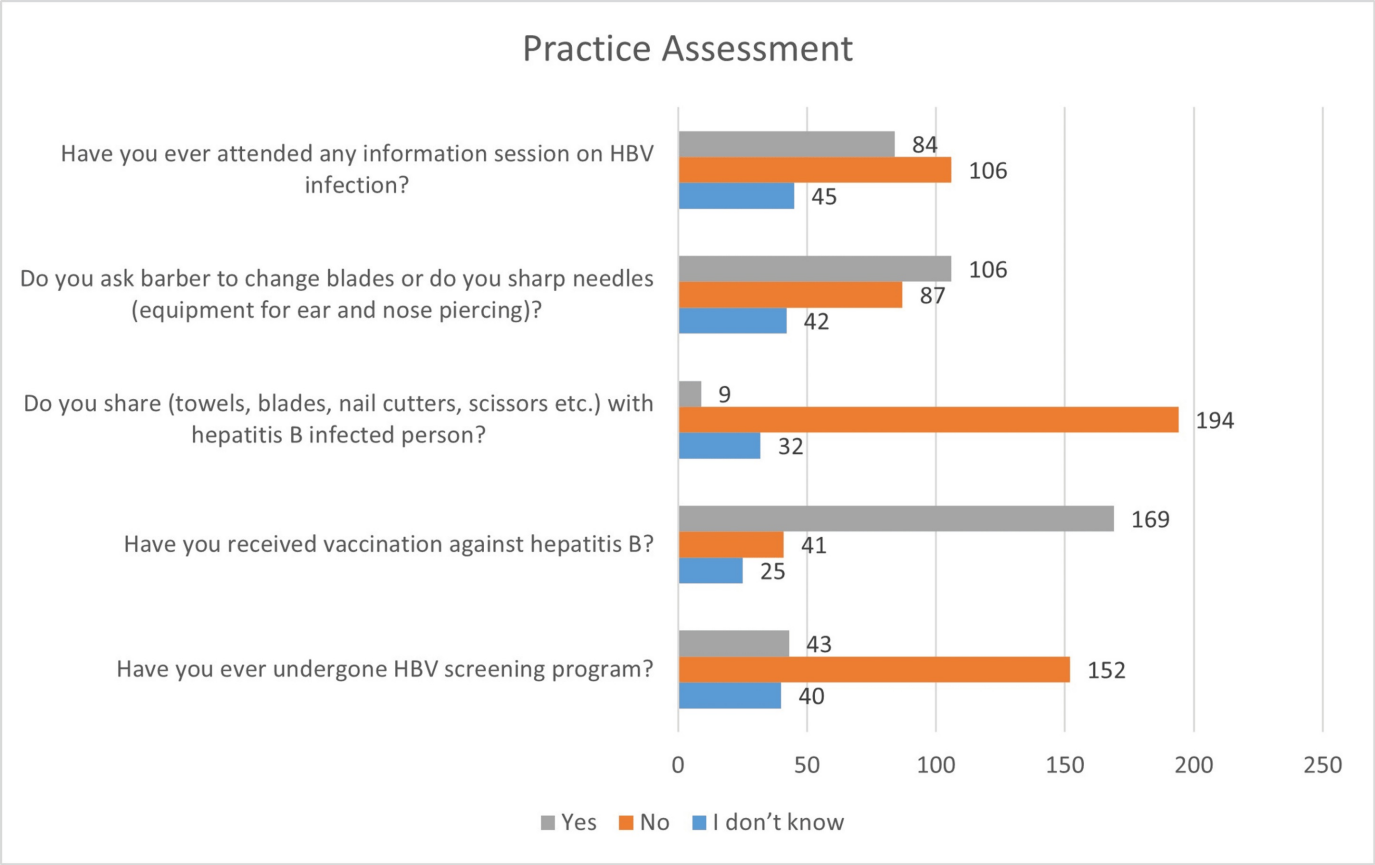
Practice assessment towards hepatitis B among participants (n=235) Data are presented as percentages (%) of participants based on selected behaviors (n=235).

Regarding HBV screening, 18.3% (n=43) had undergone screening (Figure 3). A majority (71.9%, n=169) had received the HBV vaccine. Only 3.8% (n=9) admitted to sharing personal items like towels or blades with an HBV-positive individual. A total of 45.1% (n=106) reported asking barbers to change blades or sterilize tools. Finally, 35.7% (n=84) had attended an HBV awareness session.

KAP score, categories, and associations

The data for the knowledge score, attitude score, and practice score were not normally distributed. Consequently, the median and interquartile range (IQR) were used to describe the central tendency and dispersion of the scores (Table 5).

**Table 5:** Scores of knowledge, attitude, and practice of participants (n=235) Data are presented as medians and interquartile ranges (IQR) due to non-normal distribution.

Variable	Median (IQR)	Minimum	Maximum
Knowledge Score	11.00 (9.00-13.00)	4.00	15.00
Attitude Score	15.00 (14.00-17.00)	7.00	21.00
Practice Score	2.00 (2.00-3.00)	0.00	5.00

**Figure 4:**
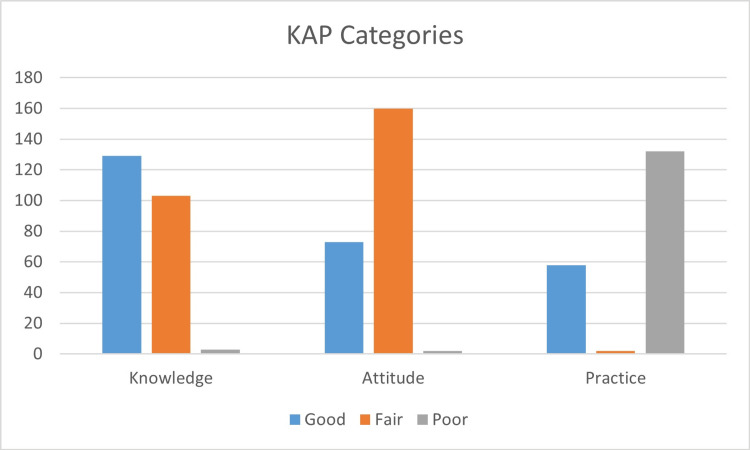
Knowledge, attitude, and practice (KAP) towards hepatitis B categories among participants (n=235) Participants categorized by KAP levels. Data shown as frequencies (N) and percentages (%).

The distribution of participants across different levels of KAP revealed that the majority demonstrated good knowledge (54.9%, n=129) and fair attitude (68.1%, n=160), while a significant portion exhibited poor practice (56.2%, n=132) (Figure 4).

Knowledge levels varied significantly by age (p=0.007), occupation (p<0.001), and source of information (p=0.014), but not by gender (p=0.952). Attitude levels differed significantly by age (p=0.034), occupation (p<0.001), and source of information (p<0.001), but not by gender (p=0.878). Practice levels were significantly different by gender (p=0.033) and occupation (p=0.003), but not by age (p=0.904) or source of information (p=0.772). Significant positive correlations were found between attitude and knowledge (p<0.001), practice and knowledge (p=0.001), and practice and attitude (p<0.001). Table 6 presents the association between KAP levels and demographic variables among participants.

**Table 6:** Association between knowledge, attitude, and practice levels and demographic variables among participants (n=235) Values shown are raw frequencies for good/fair/poor KAP levels. Test used: chi-square (χ²). p<0.05 considered statistically significant.

Variable	Category	Knowledge (G/F/P)	Attitude (G/F/P)	Practice (G/F/P)	χ² value	p-value
Age	17–20	30/48/14	6/70/16	5/25/62	9.76	0.007
	21–23	80/40/9	25/92/12	10/35/84	8.53	0.034
	24–26	19/4/1	3/4/7	5/18/1	0.63	0.904
Gender	Male	65/40/10	9/88/18	15/35/65	0.004	0.952
	Female	64/46/10	25/72/23	10/43/67	4.54	0.033
Year of Study	3rd Year	35/35/8	3/64/11	6/22/50	18.67	<0.001
	4th Year	40/30/8	10/60/8	8/25/45	17.02	<0.001
	5th Year	54/21/4	21/36/22	11/31/37	11.65	0.003
Info Source	HC Workers	70/30/8	18/75/15	10/30/73	8.55	0.014
	Posters	3/4/2	0/8/1	0/4/5	19.87	<0.001
	Others	30/35/8	10/50/14	5/22/46	1.12	0.772
	TV/Internet	26/17/3	6/27/11	10/18/16		

## Discussion

Promoting awareness is a cornerstone of any strategy to control and prevent infectious diseases. Medical students are at the forefront of healthcare provision, and it is expected that they know the routes of transmission of different infectious agents to protect their patients and themselves from nosocomial infections.

This study revealed that the overall knowledge regarding HBV infection, prevalence, and the mode of transmission is high among medical students who participated in the study, with a majority of them expressing a high knowledge regarding the main mode of transmission of HBV infection. Conversely, there were misconceptions about some mode of transmission where medical students expressed moderate knowledge regarding the fact that eating and drinking utensils do not transmit hepatitis B. This was in line with what was obtained from the study done in Bamenda Health District, NWR, Cameroon, among healthcare workers. However, this contradicted other studies done in Ethiopia [9], the Lao Democratic People’s Republic (Lao DPR) [10], India [11], and Malaysia [12], which revealed poor knowledge about HBV infection. This may be justified by the fact that medical education trains individuals to acquire, evaluate, and use information accurately [12].

Most participants in this study had a good knowledge regarding the possibility of transmission of HBV infection by asymptomatic, but positive HBsAg patients, although knowledge concerning whether chronic HBV is curable was poor.

This is similar to what was obtained from the study done in Bamenda Health District, NWR, Cameroon, among healthcare workers who reported high knowledge of all aspects of the modes of transmission of HBV. However, this contradicted previous studies carried out in the Lao DPR [10], India [11], and Malaysia [12] in which very poor knowledge of participants was reported. This fact might be attributed to an adequate virology curriculum and sufficient clinical training in interpreting test results, which were available for the medical students in this study.

The majority of participants in this study expressed positive attitudes towards HBV infection, with the need for potential social isolation for those with HBV infection and encouragement of early treatment upon diagnosis. The majority also acknowledged the effectiveness of the HBV vaccine. This was again similar to what was obtained from the study carried out in Bamenda Health District, NWR, Cameroon, among healthcare workers, who showed an overall positive attitude towards HBV. However, this completely contradicted the findings of the studies carried out in Tanzania, Lao DPR [10], and Malaysia [13-14] in which poor attitudes towards HBV infection were detected. This may be explained by the fact that developing a positive attitude towards a disease is generally associated with acquiring adequate knowledge on that disease.

Participants in this study showed a very good practice towards HBV infection, with the majority of the participants reporting receiving full doses of HBV vaccine and avoiding sharing personal items such as towels, blades, and nail cutters with an HBV-infected person. Overall, the practice adopted by the participants was very good. This was in line with the results obtained from the study carried out in Bamenda Health District, NWR, Cameroon, among healthcare workers [15], who reported a good practice towards HBV infection and contradicted other results from studies carried out in Ethiopia [9] and Tanzania [16,17].

This could easily be anticipated, given that better knowledge of the route of disease transmission should help the individual take precautions against getting an infection.

In our study, we noticed that having more clinical years spent as trainees in hospitals, as well as having encountered HBV patients, was related to higher levels of KAP achieved.

Limitations and strengths

The main limitations of this study were the fact that vaccination status was self-reported and not confirmed by the measurement of the anti-hepatitis B surface antibody (HBsAb) titer of the students. Therefore, recall bias and erroneous information might have affected the findings of the study. Again, since the study was conducted only at UMST among third- to fifth-year students, the results cannot be generalized to all medical colleges in the Sudan, but they will undoubtedly serve as a background for future studies in the region. In addition, the design of the study was cross-sectional, and it did not measure the cause-and-effect relationship. Finally, it is necessary to implement enlightenment programs to improve students’ KAP, towards HBV infection, particularly in weak areas. In addition, we recommend that future studies explore students’ HBV test and immunization statuses in terms of their associations with HBV KAP levels.

## Conclusions

In conclusion, the level of knowledge regarding HBV transmission among medical students was generally fair, though misconceptions were present - just over half of the participants correctly identified that sharing eating and drinking utensils does not transmit HBV. There was also limited consensus about the curability of hepatitis B, with slightly less than half of the participants mistakenly believing the disease is curable, possibly due to an inadequate understanding of HBV seroconversion. Nonetheless, most participants demonstrated good knowledge of HBV symptoms. Attitudes towards HBV were positive overall, with a considerable number of students recognizing their occupational risk, supporting early treatment upon diagnosis, and expressing confidence in the protective role of the HBV vaccine. Participants also displayed appropriate social attitudes, supporting measures such as limited isolation when necessary. Practice levels were moderate; while most students reported personal precautions such as requesting barbers to change blades or ensuring sterile conditions for piercings, only about one-third had attended an HBV information session. Given their heightened exposure to blood and bodily fluids, all medical students should be vaccinated against HBV upon admission. The COVID-19 pandemic has disrupted healthcare systems and educational programs in Sudan, possibly contributing to the moderate vaccination coverage observed. Structured interventions - such as pretesting, educational lectures, hands-on demonstrations, and post-testing - can significantly improve knowledge and foster attitudinal change. Therefore, we recommend the implementation of mandatory vaccination programs and the integration of structured infection prevention training, including workshops and lectures, to enhance medical students' knowledge, attitude, and practice towards HBV prevention and control.

## Appendices

Hepatitis B knowledge, attitude, and practice questionnaire

**Table 7:** Demographic profile of medical student participants

Part I - Demographic characteristics	
1. Age	Please enter your age in a numeric form
2. Gender	□ Male □ Female
3. Year of Study	□ 3rd Year □ 4th Year □ 5th Year

**Table 8:** Knowledge assessment items on hepatitis B infection

Part II - Knowledge	
4. Do you have any information about hepatitis B?	□ Yes □ No
5. Source of information	□ TV/Radio/Internet □ Health-care workers □ Posters □ Others
6. Is hepatitis B caused by a virus?	□ Yes □ No □ Don’t know
7. Modes of transmission	Contaminated blood, Sharing needles, Shaking hands, Used blades, Sneezing, Sharing food, Mother-to-child, Unsafe sex, Water, Don’t know
8. Symptoms	Excessive hunger, Sweating, Vomiting, Abdominal pain, Excessive talking, Nausea, Jaundice, Loss of appetite, Don’t know
9. Can hepatitis B cause liver cancer?	□ Yes □ No □ Don’t know
10. Is hepatitis B curable?	□ Yes □ No □ Don’t know
11. Is there a vaccine?	□ Yes □ No □ Don’t know
12. Can asymptomatic HBV-positive people transmit HBV?	□ Yes □ No □ Don’t know
13. Can sharing utensils transmit hepatitis B?	□ Yes □ No □ Don’t know

**Table 9:** Attitudinal statements on hepatitis B and preventive behavior

Part III - Attitude	
14. I am not at risk of getting HBV infection	□ Strongly Disagree □ Disagree □ Agree □ Strongly Agree
15. A person positive for hepatitis B may face isolation	□ Strongly Disagree □ Disagree □ Agree □ Strongly Agree
16. I would consult a doctor if I had HBV symptoms	□ Strongly Disagree □ Disagree □ Agree □ Strongly Agree
17. Early treatment is better than waiting for symptoms	□Strongly Disagree □ Disagree □ Agree □ Strongly Agree
18. HBV treatment is expensive and unaffordable	□ Strongly Disagree □ Disagree □ Agree □ Strongly Agree
19. HBV vaccine helps prevent infection	□ Strongly Disagree □ Disagree □ Agree □ Strongly Agree
20. In married couples, one partner won’t transmit HBV to the other	□ Strongly Disagree □ Disagree □ Agree □ Strongly Agree
21. HBV is not a major community concern	□Strongly Disagree □ Disagree □ Agree □ Strongly Agree

**Table 10:** Self-reported practices related to hepatitis B prevention

Part IV - Practice	
22. Have you undergone HBV screening?	□ Yes □ No □ Don’t know
23. Have you received the HBV vaccine?	□ Yes □ No □ Don’t know
24. Do you share personal items with HBV-positive individuals?	□ Yes □ No □ Don’t know
25. Do you ask barbers to change blades?	□ Yes □ No □ Don’t know
26. Have you attended an HBV information session?	□ Yes □ No □ Don’t know
